# Enhanced Sensing Ability of Brush-Like Fe_2_O_3_-ZnO Nanostructures towards NO_2_ Gas via Manipulating Material Synergistic Effect

**DOI:** 10.3390/ijms22136884

**Published:** 2021-06-26

**Authors:** Yuan-Chang Liang, Yu-Wei Hsu

**Affiliations:** Department of Optoelectronics and Materials Technology, National Taiwan Ocean University, Keelung 20224, Taiwan; david4250557@gmail.com

**Keywords:** synthesis, microstructure, composite, sensing ability, enhanced mechanism

## Abstract

Brush-like α-Fe_2_O_3_–ZnO heterostructures were synthesized through a sputtering ZnO seed-assisted hydrothermal growth method. The resulting heterostructures consisted of α-Fe_2_O_3_ rod templates and ZnO branched crystals with an average diameter of approximately 12 nm and length of 25 nm. The gas-sensing results demonstrated that the α-Fe_2_O_3_–ZnO heterostructure-based sensor exhibited excellent sensitivity, selectivity, and stability toward low-concentration NO_2_ gas at an optimal temperature of 300 °C. The α-Fe_2_O_3_–ZnO sensor, in particular, demonstrated substantially higher sensitivity compared with pristine α-Fe_2_O_3_, along with faster response and recovery speeds under similar test conditions. An appropriate material synergic effect accounts for the considerable enhancement in the NO_2_ gas-sensing performance of the α-Fe_2_O_3_–ZnO heterostructures.

## 1. Introduction

Increasing awareness of harmful gases as an environmental problem has resulted in the vital improvement of gas sensor technology. Especially, NO_2_ gas from fuel and vehicle exhausts, even if the concentration is low, is hazardous to the human body and the ecosystem. The design and development of various semiconductors with diverse architectures to detect NO_2_ gas molecules with high efficiency are in high demand in material technology. Hematite (α-Fe_2_O_3_) and zinc oxide (ZnO) are n-type semiconductors widely researched in gas sensor applications owing to their superior stability, low cost, and preparation simplicity [1,2]. Approaches to synthesizing low-dimensional α-Fe_2_O_3_ and ZnO structures are diverse, and they both exhibit potent sensitivity toward various volatile and toxic gases [1,2,3,4,5]. Notably, a large-scale growth and low-cost chemical hydrothermal method has been proposed to prepare low-degree α-Fe_2_O_3_ and ZnO materials for the high reproducibility and uniformity of sample preparation [2,3]. Despite the encouraging development of low-degree α-Fe_2_O_3_ and ZnO nanostructures, the improvement of their gas sensitivity and selectivity to target gases remains a challenge. Considerable attention has been paid to the synthesis of oxide heterostructures. Promising research into oxide heterostructures consisting of Fe_2_O_3_ and ZnO has indicated that a Fe_2_O_3_–ZnO composite system can improve the detection ability toward various harmful gases in comparison with that of single-constituent counterparts. However, most work on Fe_2_O_3_–ZnO heterostructures has been conducted on independent variables to improve their gas sensitivity; for example, controlling the thickness of the decorated ZnO shell for comparison with its Debye length [6], and controlling the geometrical shape of the composite structure [7]. How to integrate multiple factors for the synergetic optimal gas sensitivity of Fe_2_O_3_–ZnO heterostructures is still a scientific concern in this research field. Notably, low-concentration NO_2_ gas emitted from fuel and vehicles and chemical plants threatens the environment and human health and safety. Studies on the NO_2_ gas detection capabilities and effective sensing mechanism of Fe_2_O_3_–ZnO composite-based sensors are limited. In this study, the low-concentration NO_2_ gas-sensing behavior and mechanism of a brush-like α-Fe_2_O_3_–ZnO heterostructure synthesized through an integrated sputtering ZnO seed-assisted hydrothermal growth method were investigated. The substantially improved low-concentration NO_2_ gas-sensing performance of the Fe_2_O_3_ nanorod template through branched ZnO decoration was achieved based on the control of proper material synergistic effects, including the surface morphology, surface area, heterogeneous barrier, and the Debye length of branched crystals. The material design and fundamental synergetic effects on the NO_2_ gas-sensing characteristic of brush-like Fe_2_O_3_–ZnO heterostructures are discussed.

## 2. Results

### 2.1. Microstructural Analysis

**Figure 1:**
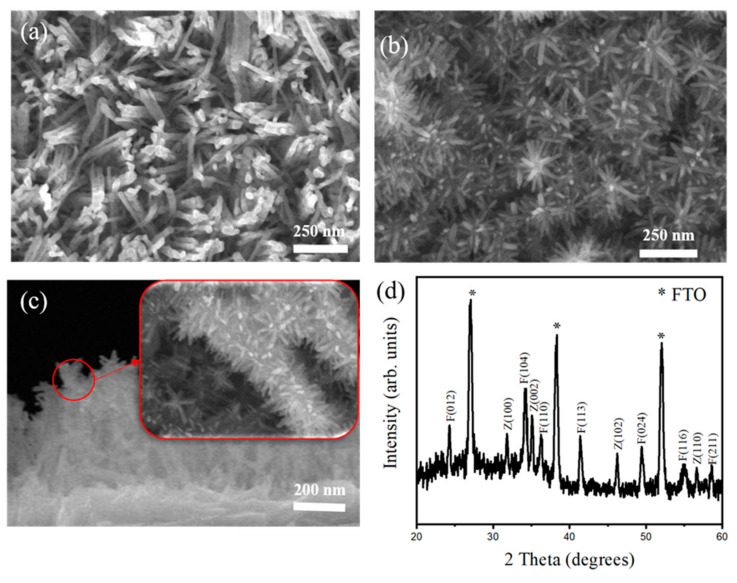
SEM top view image: (**a**) pristine Fe_2_O_3_ nanorod template and (**b**) Fe_2_O_3_–ZnO. (**c**) Cross-section SEM image of Figure (**b**) and the subfigure is an enlarged image. (**d**) XRD pattern of Fe_2_O_3_–ZnO heterostructures. The asterisk, Z, and F denote FTO, ZnO, and Fe_2_O_3_, respectively.

Figure 1a presents a scanning electron microscopic image of pristine α-Fe_2_O_3_ nanorods grown vertically aligned on the substrate. The average diameter of the nanorods was approximately 50 nm, with lengths of several micrometers. Figure 1b,c illustrate the morphology of as-synthesized Fe_2_O_3_–ZnO nanostructures. The surface of the Fe_2_O_3_–ZnO heterostructure was built from compactly aggregated tiny rods in the branch structures of the brush-like Fe_2_O_3_–ZnO heterostructures. Comparatively, the surface of the pristine Fe_2_O_3_ nanorods was smooth, and the as-synthesized brush heterostructures had more applicable surface area. The brush Fe_2_O_3_–ZnO heterostructures are more suitable for gas sensor application than individual component nanostructures are because of the rough, abundant pipes between branches and well-aligned surfaces, including the stem and branch parts of the heterostructures. Figure 1d presents the XRD patterns of the as-prepared Fe_2_O_3_–ZnO heterostructures. Diffraction peaks of α-Fe_2_O_3_ and well-defined diffraction peaks associated with hexagonal ZnO appeared in the XRD pattern according to the reference JCPDS card data in rhombohedral α-Fe_2_O_3_ JCPDS card (No. 33-0664) and hexagonal ZnO (No. 36-1451), respectively. Notably, the ZnO (002) is the most intensive ZnO crystallographic plane, which is in agreement with the crystallographic feature of the ZnO nanorods grown along its *c*-axis direction, synthesized via several approaches [7,8]. The XRD result proves the good crystalline quality of the Fe_2_O_3_–ZnO heterostructures herein.

### 2.2. UV–vis Optical Characterization

**Figure 2:**
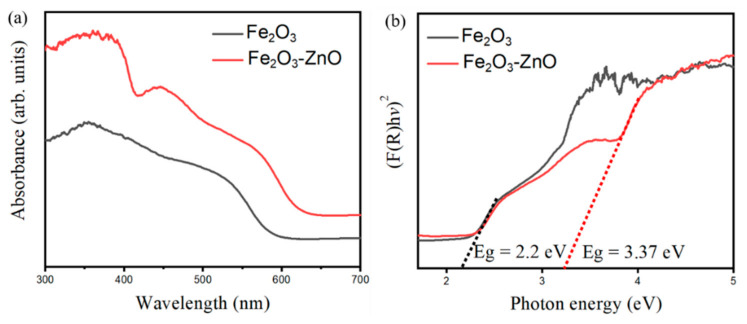
UV–vis absorption spectra of (**a**) pristine Fe_2_O_3_ nanorod template and Fe_2_O_3_–ZnO. (**b**) Evaluation of bandgap energies of Fe_2_O_3_ and Fe_2_O_3_–ZnO.

The optical properties of the as-synthesized samples were investigated by recording UV–vis absorbance spectra. Figure 2a shows the UV–Vis absorbance spectra of pristine Fe_2_O_3_ and a Fe_2_O_3_–ZnO composite. It is evident that a steep absorption edge appears at the visible light region of approximately 500–600 nm for the Fe_2_O_3_. This is attributed to the band-to-band transition in the α-Fe_2_O_3_ phase [9] Notably, the optical absorbance spectrum of the Fe_2_O_3_–ZnO has two obvious step absorption edges located at UV and visible light regions. The appearance of the absorption edge at the UV region is associated with the decorated ZnO, which has an UV region band energy feature [10]. The analysis of the optical absorbance spectra herein demonstrates the Fe_2_O_3_–ZnO heterostructure has successfully constructed through multiple hydrothermal routes with the assistance of sputtering ZnO seed layer engineering. The bandgap energies of the Fe_2_O_3_ and ZnO are also evaluated by converting the Kubelka−Munk equation, as displayed in Figure 2b [11]. From Figure 2b, the Fe_2_O_3_ and ZnO have band gap energies of approximately 2.2 eV and 3.37 eV, respectively; this bandgap energy information was further used to construct the band diagram for discussing the gas-sensing mechanism of the as-synthesized composite.

### 2.3. Transmission Electron Microscopy Analysis

**Figure 3:**
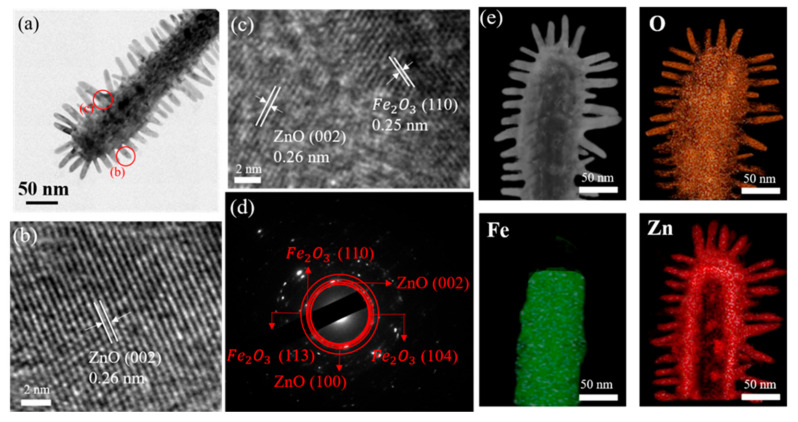
TEM image of Fe_2_O_3_–ZnO heterostructure: (**a**) low-magnification image. (**b**,**c**) Local HRTEM images of the heterostructure. (**d**) SEAD pattern taken from several composites. (**e**) EDS elemental mapping analysis of an individual Fe_2_O_3_–ZnO heterostructure.

Figure 3a presents the TEM image of a Fe_2_O_3_–ZnO heterostructure. A good brush structure was visibly displayed, and the branched rods had an average diameter and length of 12 nm and 25 nm, respectively. Figure 3b,c display the HRTEM images of the heterostructure taken from various local regions. The ordered lattice fringes with interval distances of 0.28 nm and 0.26 nm were corresponded to the interplanar spacings of α-Fe_2_O_3_ (110) and ZnO (002), respectively. Figure 3d demonstrates the selected area electron diffraction (SAED) pattern taken from several Fe_2_O_3_–ZnO composites, revealing that the concentric rings could be attributed to α-Fe_2_O_3_ (104), (110), and (113) and the ZnO (100), (002). The SAED analysis responds to the XRD result, confirming that crystalline Fe_2_O_3_–ZnO heterostructures were successfully formed. Figure 3e shows the energy dispersive spectroscopy (EDS) elemental mapping analysis of an individual Fe_2_O_3_–ZnO heterostructure. The Fe signals could be only detected in the core region, while Zn signals were predominant in the outer region, and the distribution of Zn element further confirmed that the surface of the Fe_2_O_3_ was decorated by ZnO nanorods. The O signals can be recognized from the whole heterostructure. The EDS mapping analyses confirmed that the as-synthesized product formed a compositionally homogeneous composite.

### 2.4. Surface Active Site Analysis

**Figure 4:**
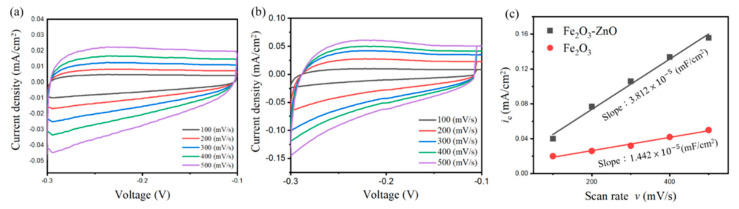
(**a**) Cyclic voltammetry plot of pristine Fe_2_O_3_ and (**b**) Fe_2_O_3_–ZnO at different scan rates. (**c**) ECSA analysis of Fe_2_O_3_ and Fe_2_O_3_–ZnO.

Surface area is an important factor that affects the gas sensing characteristic, which should be investigated for the heterostructure sample. The electrochemical catalyst surface activity (ECSA) of the as-fabricated α-Fe_2_O_3_ and Fe_2_O_3_–ZnO heterostructure sample are estimated with the cyclic voltammetry (CV) from the double-layer capacitance (C_dl_). As shown in Figure 4a,b, the CV curves of α-Fe_2_O_3_ and Fe_2_O_3_–ZnO, respectively, at non-Faradaic potential regions (−0.3–0.1 V vs. NHE) separated with different scan rates, from 0.1 mV/s to 0.5 mV/s. The ECSA can be calculated from the C_dl_ divided by the specific capacitance (C_s_), namely ECSA = C_dl_/C_s_, and is proportional to the C_dl_ value. The correlation between the double-layer capacitance (C_dl_) and the double-layer charging current (*i*_c_) follows the equation: C_dl_ = *i*_c_ /ν. The *i*_c_ = (j_a_ − j_c_), in which j_a_ is anodic current and j_c_ is cathodic current at the middle potential (−0.2 V) against the CV scan rate. Figure 4c shows that the plot of *i*_c_ vs. ν has a linear characteristic and the slope corresponds to the C_dl_. Notably, for a comparison, the *i*_c_ vs. ν plot of the pristine Fe_2_O_3_ was also included in Figure 4c. Obviously, the Fe_2_O_3_–ZnO has a larger ECSA size, which is approximately 3.8 times higher than that of the pristine Fe_2_O_3_. The larger ESCA provided more active sites for the interface reaction between photoelectrode and electrolyte, and this is attributable to higher diffusion space between the numerous tiny branched ZnO nanorods of the heterostructure, as confirmed by the aforementioned structural analysis [12,13]. The ECSA analysis reveals that the brush heterostructure produced more surface area for reaction species adsorb or desorb on it, which might further benefit its gas-sensing ability.

### 2.5. Gas Sensing Performance

**Figure 5:**
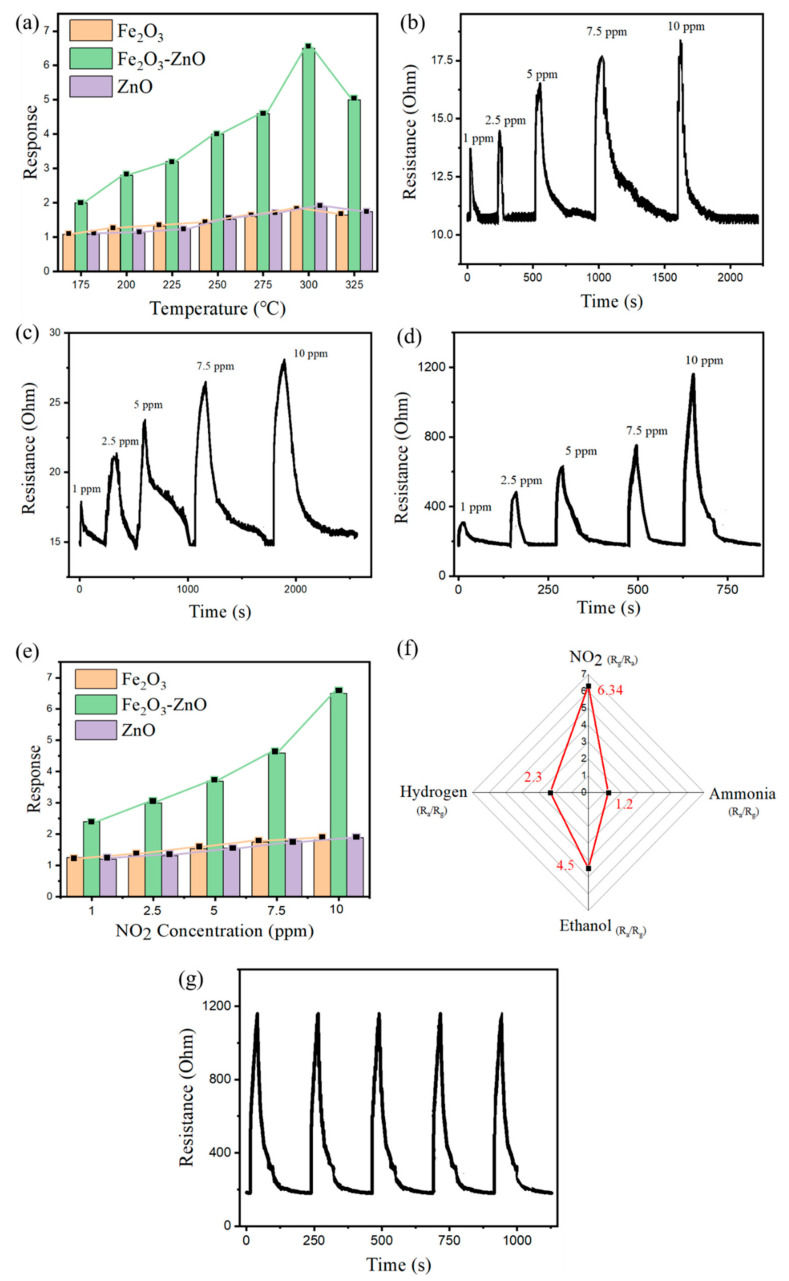
(**a**) Operating temperature-dependent 10 ppm NO_2_ gas-sensing response for Fe_2_O_3_, ZnO, and Fe_2_O_3_-ZnO sensors. (**b**–**d**) Dynamic gas-sensing curves of Fe_2_O_3_, ZnO, and Fe_2_O_3_-ZnO sensors towards different NO_2_ gas concentrations at 300 °C, respectively. (**e**) Gas-sensing response vs. NO_2_ gas concentration for various sensors at 300 °C. (**f**) Cross-selectivity of Fe_2_O_3_-ZnO sensor. The responses are marked with red. (**g**) Cycling gas-sensing curves for the Fe_2_O_3_-ZnO sensor towards 10 ppm NO_2_ gas.

As presented in Figure 5a, the pristine Fe_2_O_3_ and Fe_2_O_3_–ZnO heterostructure-based gas-sensing responses toward 10 ppm NO_2_, as a function of the operating temperature, were studied. The dependence of gas-sensing responses versus operating temperature was the same for both gas sensors. A resultant equilibrium between the sensor material surface reaction with NO_2_ gas molecules and the diffusion of these molecules to the material surface occurred at 300 °C [14]. Figure 5b,d present the dynamic response and recovery curves of the pristine Fe_2_O_3_, ZnO, and Fe_2_O_3_–ZnO sensors toward different NO_2_ gas concentrations at 300 °C. Concentration-dependent cycling test curves for both sensors demonstrated that the Fe_2_O_3_ and Fe_2_O_3_–ZnO sensors were effective and suitably sensitive to NO_2_ at various concentrations. The gas-sensing responses of the Fe_2_O_3_ sensor to 1, 2.5, 5, 7.5, and 10 ppm NO_2_ were 1.26, 1.36, 1.54, 1.67, and 1.71, respectively. The responses of ZnO to1, 2.5, 5, 7.5, and 10 ppm NO_2_ were 1.19, 1.42, 1.59, 1.75, and 1.83. Moreover, the sensing responses of the Fe_2_O_3_–ZnO sensor to 1, 2.5, 5, 7.5, and 10 ppm NO_2_ were 1.59, 2.38, 3.17, 3.97, and 6.34, respectively. The summarized responses versus NO_2_ concentration results in Figure 5e indicate that the Fe_2_O_3_–ZnO sensor exhibits a higher gas-sensing response than that of the Fe_2_O_3_ sensor under the same test conditions, demonstrating the benefit of ZnO decoration on Fe_2_O_3_. Furthermore, these two sensors exhibited different response and recovery speeds to NO_2_ gas. The response speeds of the Fe_2_O_3_ sensor ranged 23 to 62 s for 1 to 10 ppm NO_2_; those of the Fe_2_O_3_–ZnO ranged 15 to 26 s for 1 to 10 ppm NO_2_. The Fe_2_O_3_ recovery speeds were approximately 180 to 600 s for 1 to 10 ppm NO_2_, and those of the Fe_2_O_3_–ZnO were 85 to 185 s for 1 to 10 ppm NO_2_. Substantially increased NO_2_ gas-sensing responses and recovery speeds were observed for the Fe_2_O_3_ template decorated with abundant ZnO nanorods. The formation of the unique brush-like morphology of the Fe_2_O_3_–ZnO composite substantially improved the response and recovery speeds in comparison to the Fe_2_O_3_ sensor. The improved specific surface area and numerous heterojunctions of the Fe_2_O_3_−ZnO composite accounted for the observed results. A similar combined effect of hierarchical morphology and heterojunction that enhances gas-sensing performance has been proposed in an Fe_2_O_3_–TiO_2_ composite system towards trimethylamine gas [15]. For practical usage, a gas sensor with selectivity must detect a target gas when exposed to a multicomponent gas environment; high sensor reliability within the sensing environment is a key concern. Figure 5f presents the cross-sensitivity of the Fe_2_O_3_–ZnO observed upon exposure to 10 ppm NO_2_, 100 ppm ethanol vapor, 100 ppm ammonia, and 100 ppm hydrogen gas at 300 °C. When the comparative target gases of ethanol vapor, ammonia gas, and hydrogen gas had a higher concentration of 100 ppm, the sensing responses of the Fe_2_O_3_–ZnO sensor to these gases were still substantially lower than the response to 10 ppm of NO_2_, revealing the superior gas-sensing selectivity of Fe_2_O_3_–ZnO toward NO_2_ gas. The reasons for the selectivity of oxide sensing materials toward a specific target gas are complicated and no consistent statements are proposed in the literature. It is proposed that that the chemical properties of various oxidizing or reducing gases could cause a difference in the adsorption ability and reaction strength with adsorbed O species at the given sensor’s operating temperature [16]. Therefore, higher selectivity toward NO_2_ gas for the Fe_2_O_3_–ZnO herein might be associated with the higher electron affinity of NO_2_ gas (2.28 eV) as compared with pre-adsorbed oxygen (0.43 eV) and other test gases [17]. The unpaired electron in the N atom of the NO_2_ gas molecule easily forms bond with the oxygen species presented on the Fe_2_O_3_–ZnO surface, which increases the chemisorption size of NO_2_ as compared to other target gases. Figure 5g presents the cycling gas-sensing curves for the Fe_2_O_3_–ZnO sensor to 10 ppm NO_2_, with no clear sensing response deterioration after five cycling tests, demonstrating the stability and reproducibility of the Fe_2_O_3_–ZnO sensor. Furthermore, as exhibited in Table 1, most NO_2_ gas sensors have an operating temperature above 300 °C to obtain detectable responses. Based on the NO_2_ concentration and gas-sensing responses of various sensors in Table 1, the as-synthesized α-Fe_2_O_3_–ZnO composite sensor exhibits decent gas-sensing performance among various reported Fe_2_O_3_-based or ZnO-based composite sensors.

## 3. Discussion

**Figure 6:**
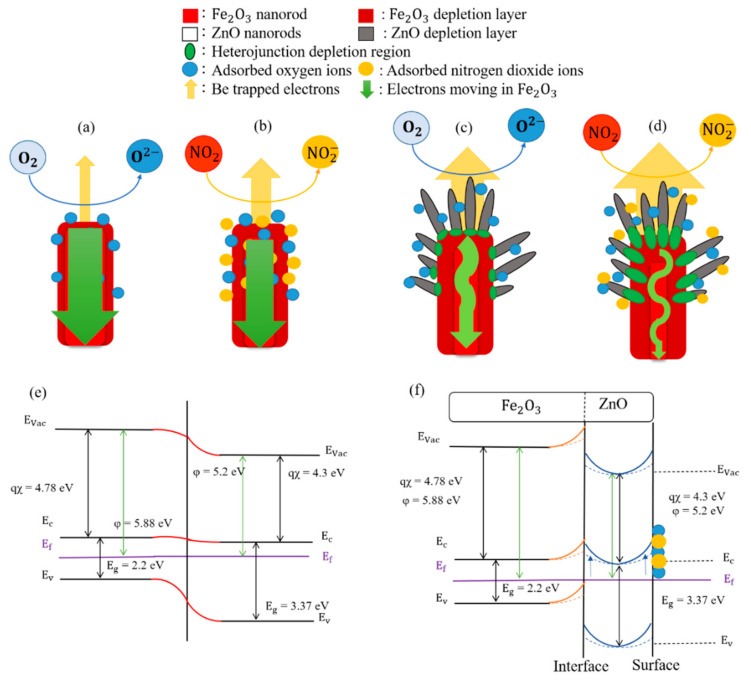
Schematic diagrams illustrating the gas sensing mechanisms of Fe_2_O_3_ template: (**a**) in air, (**b**) in NO_2_ gas, and those of Fe_2_O_3_-ZnO: (**c**) in air, (**d**) in NO_2_ gas. (**e**) Energy band diagram of Fe_2_O_3_/ZnO after equilibrium. (**f**) Band bending variation of Fe_2_O_3_/ZnO toward NO_2_ gas (from dashed line to solid line).

Figure 6a indicates that the electron transport channel size inside the pristine Fe_2_O_3_ nanorod can be modulated by the surface electron depletion layer, which surrounds the nanorod’s surface. Initially formed through the adsorption of oxygen molecules on the surface, the electrons are extracted from the conduction band of the nanorods to form adsorbed oxygen ions (O_ads_^2−^) [22,23]. As presented in Figure 6b, further exposure of the Fe_2_O_3_ nanorod template to NO_2_ gas resulted in the marked extraction of its surface electrons through the surface reaction with the NO_2_ molecules, forming surface-adsorbed NO_2_^−^ ions. The conducting channel of the Fe_2_O_3_ nanorod was further narrowed, thereby increasing the bulk resistance of the Fe_2_O_3_ sensor. For the NO_2_ gas-sensing mechanism of the brush-like Fe_2_O_3_–ZnO heterostructure in Figure 6c,d, the influence of the sample morphology for highly accessible NO_2_ gas molecule adsorption should be considered. The appreciable morphological difference between the composite and pristine Fe_2_O_3_ may result in several concomitant effects in the composite, such as high reactive surface area size, high airflow channel effect, and the high surface trapping and low reaction time with the target gas molecules. The aforementioned ECSA result demonstrated the substantially enhanced surface reaction of the Fe_2_O_3_–ZnO compared with that of the pristine Fe_2_O_3_. The brush-like configuration is also beneficial in the formation of pathways between the branches. The target gas molecules can effectively react with the sample surface, increasing the number of captured electrons from the Fe_2_O_3_ and ZnO conduction bands and effectively reducing the response and recovery time during gas-sensing tests [24,25]. The diameter of decorated ZnO branches plays a vital role in the gas-sensing performance of the heterostructure. The average diameter of the ZnO branches was approximately 12 nm, and the Debye length of the ZnO at increased temperatures was approximately 20 nm [26]. The decorated ZnO nanorods should be fully depleted at the operating temperature herein. The gas-sensing response abruptly increases when the oxide semiconductor particle size becomes smaller than the Debye length [27]. Moreover, the Fe_2_O_3_–ZnO sensor’s gas-sensing response to NO_2_ gas was not only influenced by the depletion layer from the surfaces of each ZnO branch and Fe_2_O_3_ nanorod template, but also by the formation of abundant Fe_2_O_3_/ZnO heterogeneous junctions. These form because the different work functions of Fe_2_O_3_ and ZnO in the composite contribute varying degrees of bulk resistance upon exposure to NO_2_ gas [7]. The percolation network of electrons pass through potential barriers at heterogeneous junctions between the ZnO branches and Fe_2_O_3_ template. This results in high initial bulk resistance (189 Ohm) in the Fe_2_O_3_–ZnO sensor, almost 18 times that of the pristine Fe_2_O_3_ (10.5 Ohm). The initially formed Fe_2_O_3_/ZnO interfacial potential barriers effectively modulate the electron transport between the two constituents by adsorbing or desorbing target gas molecules [28]. These junctions can be considered additional active sites, resulting in the enhancement of the gas-sensing response when the sensor material is exposed to NO_2_ gas. When the Fe_2_O_3_–ZnO was further exposed to NO_2_ gas, as presented in Figure 6d, the Fe_2_O_3_/ZnO interfacial depletion layer size was appreciably widened, resulting in the rugged, narrow conduction channel of the Fe_2_O_3_ template. The band emery variations of the Fe_2_O_3_/ZnO at the surface and interface regions also account for the improved NO_2_ gas-sensing performance of the Fe_2_O_3_-ZnO composite. Figure 6e shows the band diagram of the Fe_2_O_3_/ZnO system at the equilibrium state which is constructed with the band energy parameters from previous bandgap value evaluation and literature [7]. Figure 6f displays the band structure variation of the Fe_2_O_3_-ZnO exposed to NO_2_ gas. The Fe_2_O_3_/ZnO heterogeneous interface due to the electron trapping by interface states might dominate the gas-sensing behavior of the composite toward NO_2_ gas. The potential barrier-controlled carrier transport mechanism might be predominant over the surface depletion mechanism for Fe_2_O_3_-ZnO; this is due to the fact that no electrons are available in the depleted decorated ZnO (size smaller than Debye length) to react with the NO_2_ molecules at the given test condition. Comparing the schematic configurations of NO_2_ gas-sensing mechanisms in Fe_2_O_3_ and Fe_2_O_3_-ZnO, the degree of bulk resistance variation associated with ZnO and Fe_2_O_3_ surface depletion and the Fe_2_O_3_/ZnO interfacial junctions in the heterostructural system were expected to be substantially higher than that of the pristine Fe_2_O_3_ template, which was merely modulated by the surface depletion layer size variation before and after exposure to NO_2_ gas. Based on the proposed NO_2_ gas-sensing model of the Fe_2_O_3_–ZnO heterostructure, the low-concentration NO_2_ gas-sensing performance of the pristine Fe_2_O_3_ nanorods was considerably improved through the suitable control of material synergistic effects through the formation of brush-like Fe_2_O_3_–ZnO heterostructures.

## 4. Materials and Methods

### 4.1. Materials Synthesis

The α-Fe_2_O_3_ nanorod template was synthesized through a hydrothermal method. The 3.028 g of iron chloride hexahydrate (FeCl_3_⋅6H_2_O) and 0.72 g of urea (CO(NH_2_)_2_) were dissolved into deionized water to form a 60 mL mixture solution as a reaction solution. The F-doped tin oxide (FTO) substrates were perpendicularly leant in the aforementioned solution, and then tightly sealed and maintained at 100 °C for 24 h in an electric oven. Finally, the products were annealed at 500 °C for 30 min in the muffle furnace. The detailed hydrothermal reaction parameters have also been described elsewhere [3]. The Fe_2_O_3_ nanorod template was coated with a ZnO seed layer via radio-frequency sputtering at room temperature with a ZnO ceramic target. The sputtering process was conducted in an Ar/O_2_ mixed atmosphere with an Ar/O_2_ ratio of 4/1. The sputtering working pressure was 20 mTorr and the sputtering power was ZnO 90 W in this study [10]. The sputtering duration of the ZnO seed layer is 60 min. After that, the ZnO seed layer-coated Fe_2_O_3_ nanorod template on FTO substrates was perpendicularly suspended in a homogeneous mixture solution containing 100 mL of deionized water, 0.7 g of Hexamethylenetetramine (C_6_H_12_N_4_), and 1.4869 g of Zinc Nitrate Hexahydrate (Zn(NO_3_)_2_). The hydrothermal reaction was conducted at 95 °C for 1.5 h to synthesize brush-like Fe_2_O_3_-ZnO samples.

### 4.2. Characterization Analysis

The surface morphology of the as-synthesized samples was investigated by scanning electron microscopy (SEM). The analysis of samples’ crystal structures was conducted by X-ray diffractometer (XRD) with Cu Kα radiation in the two theta ranges of 20–60°. Moreover, the optical absorption spectra of the samples were investigated via a diffuse-reflectance mode with an ultraviolet–visible spectrophotometer (UV–vis). Furthermore, the detailed microstructures of the composite were characterized by high-resolution transmission electron microscopy (HRTEM) equipped energy-dispersive X-ray spectroscopy (EDS). The electrochemical catalyst surface activity (ECSA) was performed in a three-electrode electrochemical system, where the as-synthesized sample was used as the working electrode, a Pt wire was used as the counter electrode, and an Ag/AgCl electrode was used as the reference electrode in an aqueous solution containing 0.5 M Na_2_SO_4_.

### 4.3. Gas Sensing Experiments

The surface of gas sensors made from the pure Fe_2_O_3_ and the Fe_2_O_3_-ZnO composite were coated with patterned platinum film for electric contacts with probes during gas-sensing measurements. Various concentrations of NO_2_ gas (1~10 ppm) were introduced into the test chamber, using dry air as the carrier gas. A resistance meter was used to observe the resistance changes of the sensor devices before and after the supply of the target gas. The responses of sensors toward NO_2_ herein are defined as Rg/Ra, Ra and Rg are the sensor resistances in air and target gas, respectively. By contrast, the responses of sensors toward other reducing gases are defined as Ra/Rg. The response and recovery speeds of sensors are defined as the duration required to reach the 90% maximum resistance toward NO_2_ gas and the duration required to decrease 90% resistance with the removal of NO_2_ gas, respectively.

## 5. Conclusions

In summary, the integrated controllable preparation of Fe_2_O_3_–ZnO brush-like hierarchical nanostructures through a combination two-step hydrothermal method with sputtering ZnO seed layer support was proposed. The NO_2_ gas-sensing performance of the Fe_2_O_3_–ZnO heterostructures was substantially improved compared with that of the pristine Fe_2_O_3_. The improved performance of the Fe_2_O_3_–ZnO sensor was attributed to synergistic effects, including a ZnO branch size smaller than the Debye length, the branched morphology of the heterostructure inducing abundant air flow channels, and the formation of abundant heterojunction barriers. The experimental results serve as a sound reference for the design of a Fe_2_O_3_–ZnO heterogeneous sensor based on the control of appropriate material synergistic effects, with the effective detection of low-concentration NO_2_ gas.

## Figures and Tables

**Table 1:** NO_2_ gas-sensing performance of various Fe_2_O_3_- and ZnO-based composites prepared using various methods [18,19,20,21].

Material	Synthesis Method	Temperature/ NO_2_ Concentration	Response	Response Time/ Recover Time (s)	Ref.
α-Fe_2_O_3_-TiO_2_	Solvothermal method	300 °C/5 ppm	2 (R_g_/R_a_)	N/A	[18]
α-Fe_2_O_3_-SnO_2_	Hydrolysis method	300 °C/1 ppm	<0.5 ((R_g_ − R_a_)/R_a_)	N/A	[19]
ZnO-Fe_2_O_3_	Co-precipitation method	400 °C/250 ppm	10.53 (R_g_/R_a_)	1000/4000	[20]
ZnO-CuO	Screen printing method	300 °C/29 ppm	5.57 (R_g_/R_a_)	N/A	[21]
α-Fe_2_O_3_-ZnO	Hydrothermal method	300 °C/10 ppm	6.34 (R_g_/R_a_)	26/185	this work

## Data Availability

The data presented in this study are available in this article.
